# Supplementary material for: Sex differences in crossover interference in house mice
Source: G3 (Bethesda). 2025 Nov 6;16(1):jkaf268. doi: 10.1093/g3journal/jkaf268 (PMC12774607; doi:10.1093/g3journal/jkaf268)
Supplement: jkaf268_Supplementary_Data [file jkaf268_supplementary_data.zip › Supplementary_tables_and_figures_G3-2025-406324.pdf]

Supplementary material

Supplementary note 1. Description of statistical models for crossover interference.

File S1. Table of 21 993 crossovers (COs) reported in (Liu *et al.* 2014). Columns are as follows.

**column**	**contents**
`funnel`	family ID (referred to as "funnel" due to shape of pedigree)
`order`	order of founder strains at the G_0 generation; this is unique for each family
`meiosis`	code identifying one of 8 meioses in the pedigree, with respect to the G_2:F_1 individual: MGM, maternal grandmother (G_1); MGF, maternal grandfather (G_1); PGM, paternal grandmother (G_1); PGF, paternal grandfather (G_1); Mf, mother of female G_2:F_1 individual (G_2); Pf, father of female G_2:F_1 individual (G_2); Mm, father of male G_2:F_1 individual (G_2); Pm, father of male G_2:F_1 individual (G_2).
`generation`	pedigree generation in which this CO occurred (G_1 or G_2)
`sex`	sex of the parent in which this CO occurred (M or F)
`chr`	chromosome on which this CO occured
`start`	physical position of proximal (left-most) marker defining this CO (bp)
`end`	physical position of distal (right-most) marker defining this CO (bp)
`from`	founder haplotype proximal to CO
`to`	founder haplotype distal to CO
`pos`	physical midpoint of CO interval (bp)
`cM`	genetic position (in cM) of this CO, on the sex-specific map

Additional notes

Physical positions are reported on the mm9/GRCm37 assembly, as this was used during the original analysis, without liftover to the current mm39 assembly. Only genetic positions are actually used in this manuscript. Founder strains are reported as 1-letter codes: A = A/J; B = C57BL/6J; C = 129S1/SvImJ; D = NOD/ShiLtJ; E = NZO/HlLtJ; F = CAST/EiJ; G = PWK/PhJ; H = WSB/EiJ.

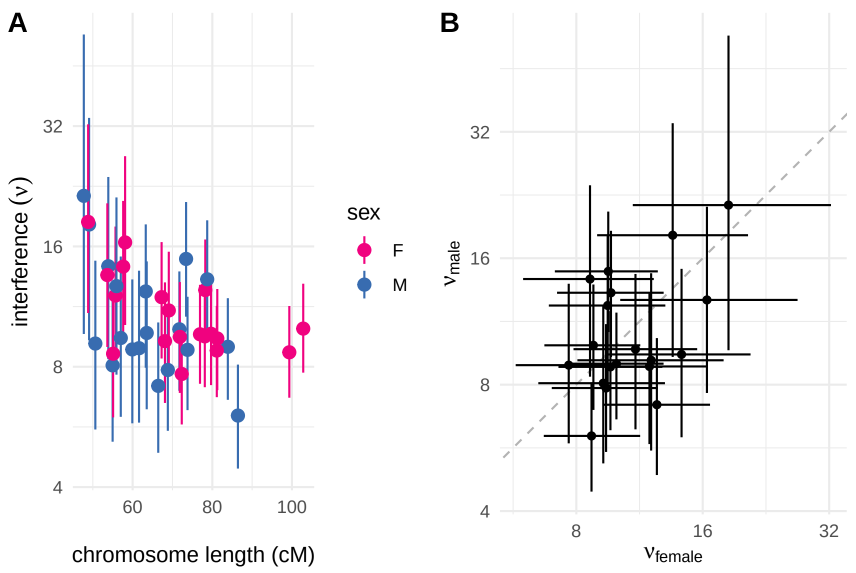

Figure S1: **Chromosome-specific interference parameters under the simple gamma model without non-interfering crossovers.** (**A**) Chromosome-specific values of ν with 95% HPDIs plotted against genetic chromosome length. (**B**) Comparison of male versus female estimates of ν for each chromosome. Dotted reference line passes through the intercept and has slope 1.

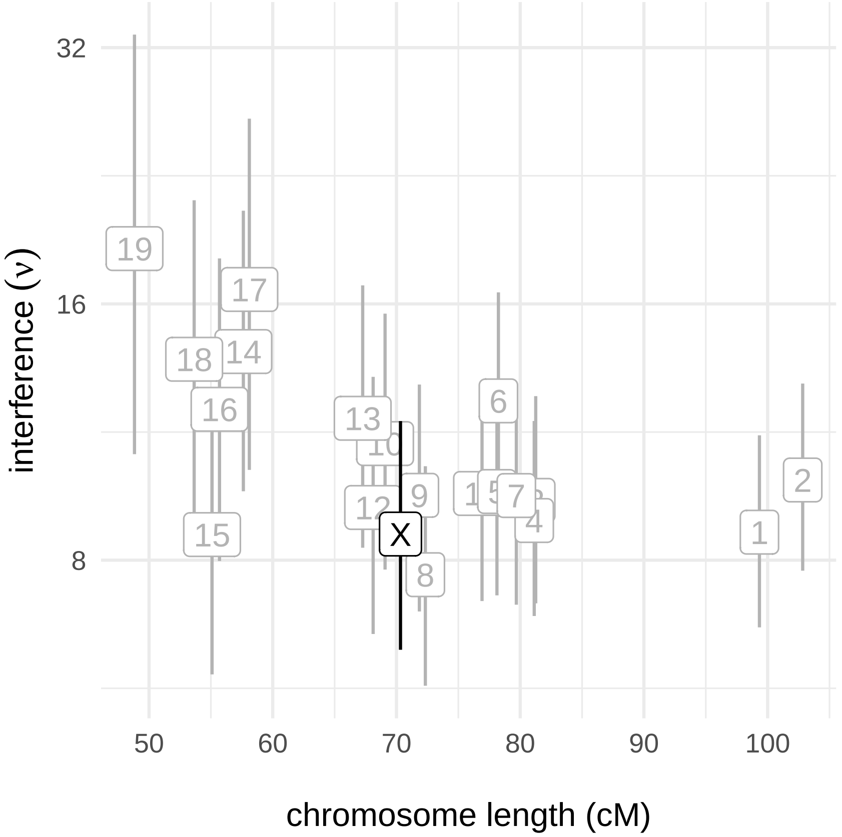

Figure S2: **Chromosome-specific interference parameters under the simple gamma model without non-interfering crossovers, in females only.** Chromosome-specific values of ν with 95% HPDIs plotted against genetic chromosome length, for labelled autosomes (grey) and the X chromosome (black).

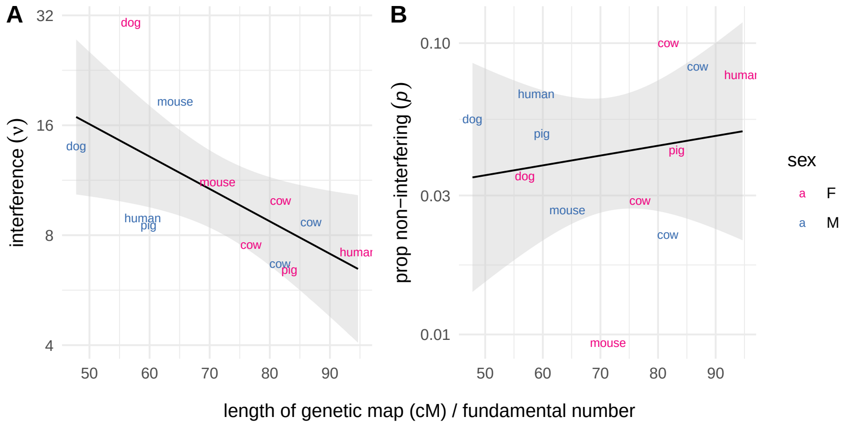

Figure S3: **Interference vs length of genetic map.** Sex-specific values of ν (**A**) and p (**B**) plotted against the average genetic length of a chromosome arm in 5 mammal species.
